# Fur functions as an activator of T6SS-mediated bacterial dominance and virulence in *Aeromonas hydrophila*

**DOI:** 10.3389/fmicb.2022.1099611

**Published:** 2023-02-08

**Authors:** Jihong Li, Zhihao Wu, Yuting Hou, Yong-An Zhang, Yang Zhou

**Affiliations:** ^1^State Key Laboratory of Agricultural Microbiology, College of Fisheries, Huazhong Agricultural University, Wuhan, China; ^2^Guangdong Laboratory for Lingnan Modern Agriculture, Guangzhou, China; ^3^Department of Microbiology and Immunology, Medical College, China Three Gorges University, Yichang, China; ^4^Shenzhen Institute of Nutrition and Health, Huazhong Agricultural University, Wuhan, China; ^5^Hubei Hongshan Laboratory, Wuhan, China; ^6^Laboratory for Marine Biology and Biotechnology, Qingdao National Laboratory for Marine Science and Technology, Qingdao, China; ^7^Engineering Research Center of Green Development for Conventional Aquatic Biological Industry in the Yangtze River Economic Belt, Ministry of Education, Wuhan, China; ^8^Shenzhen Branch, Guangdong Laboratory for Lingnan Modern Agriculture, Genome Analysis Laboratory of the Ministry of Agriculture, Agricultural Genomics Institute at Shenzhen, Chinese Academy of Agricultural Sciences, Shenzhen, China

**Keywords:** *Aeromonas hydrophila*, T6SS, fur, pathogenesis, interbacterial competition activity

## Abstract

*Aeromonas hydrophila*, a ubiquitous bacterium in aquatic habitats with broad host ranges, has earned the nickname of a ‘Jack-of-all-trades’. However, there is still a limited understanding of the mechanism of how this bacterium fit the competition with other species in dynamic surroundings. The type VI secretion system (T6SS) is macromolecular machinery found in Gram-negative bacteria’s cell envelope that is responsible for bacterial killing and/or pathogenicity toward different host cells. In this study, the depression of *A. hydrophila* T6SS under iron-limiting conditions was detected. The ferric uptake regulator (Fur) was then found to act as an activator of T6SS by directly binding to the Fur box region in *vip*A promoter in the T6SS gene cluster. The transcription of *vip*A was repressed in Δ*fur*. Moreover, the inactivation of Fur resulted in considerable defects in the interbacterial competition activity and pathogenicity of *A. hydrophila in vitro* and *in vivo*. These findings provide the first direct evidence that Fur positively regulates the expression and functional activity of T6SS in Gram-negative bacteria and will help to understand the fascinating mechanism of competitive advantage for *A. hydrophila* in different ecological niches.

## 1. Introduction

*Aeromonas*’ environmental reservoir permits it to exist at the interface of all One Health components (Lamy et al., 2021), which has garnered wide attention. *Aeromonas hydrophila*, as the most well know specie of the genus, could cause infections in a wide variety of aquatic and terrestrial animals, even possibly causing human diseases due to infections by consuming *A. hydrophila*-contaminated animals (Dong et al., 2020). Motile Aeromonas septicemia (MAS) in different fish species caused by *A. hydrophila* is economically devastating to aquaculture industries worldwide (Pridgeon and Klesius, 2011; Hossain et al., 2014). The pathogenesis of *A. hydrophila* is complicated and multifactorial, involving various virulence factors including O antigens, capsules, lipopolysaccharides (LPS), the S-layers, exotoxins, and protein secretion systems (Sha et al., 2001; Tomás, 2012; Ji et al., 2015). Protein secretion systems are required for virulence and microorganism competition.

The conserved type VI secretion system (T6SS) is a protein secretion machinery found in more than a quarter of Gram-negative pathogens (Galan and Waksman, 2018). It can be a potent virulence factor that targets host cells or a powerful weapon that targets and competes with competing bacteria in polymicrobial settings (Cianfanelli et al., 2016). The T6SS machinery is composed of 13 primary parts, with additional parts participating in effector translocation. The T6SS transenvelope complex is made up of folded Hcp protein that is covered with VgrG protein. In addition, Hcp and VgrG serve as both machine components and secreted effector proteins (Cianfanelli et al., 2016). T6SS is used by pathogens like *Vibrio cholerae*, *Pseudomonas aeruginosa*, and pathogenic *Escherichia coli* to transport multiple, diverse effector proteins into target cells. These effectors have a wide range of functions, targeting both prokaryotic and eukaryotic organisms (Hood et al., 2010; Ma et al., 2014; Unterweger et al., 2014). T6SS was discovered to contribute to *A. hydrophila*’s bactericidal activity and pathogenicity in our previous study (Li et al., 2021). T6SS appears to be tailored to the needs of each bacterium and is thus regulated by a wild range of regulatory mechanisms to ensure bacterial infection success (Leung et al., 2011). A vast range of modulators, such as the quorum-sensing system, alternative sigma factors, two-component systems, histone-like proteins (H-NS), and ferric uptake regulator (Fur), have been reported to directly or indirectly regulate the transcription of T6SSs (Bernard et al., 2011; Leung et al., 2011; Sana et al., 2012).

For most microorganisms, iron is an essential element since it is involved in many reactions in the cell. Commonly, free Fe^2+^ is rarely present in the host as it is sequestratered by several distinct cellular mechanisms (Teixido et al., 2011). Therefore, bacterial pathogens must harbor mechanisms to override iron-inhibiting defense systems in order to successfully colonize the host. Fur is a transcription factor found in many bacteria that regulates genes related to iron homeostasis and pathogenicity (Cassat and Skaar, 2013). Fur modulates gene expression by binding Fe^2+^ dependently to a specific sequence called Fur box, the consensus sequence of which is 5-GATAATGATAATCATTATC-3, situated in the regulated genes’ promoter regions (Porcheron and Dozois, 2015). It has been reported the Fur regulon contains genes involved in iron acquisition, flagellar chemotaxis, the tricarboxylic acid cycle, T3SSs, and T6SSs, and it is required for bacterial multiplication and pathogenicity (Porcheron and Dozois, 2015). Previous research shown that Fur directly suppresses T6SS expression in *Salmonella enterica* Serovar Typhimurium, *Edwardsiella tarda*, *P. aeruginosa*, and *E. coli* (Chakraborty et al., 2011; Sana et al., 2012; Ma et al., 2018; Wang et al., 2019). However, there is still limited knowledge of the function of Fur protein in *A. hydrophila*.

In this work, the expression pattern of T6SS under different iron concentrations in *A. hydrophila* was investigated. According to our findings, Fur binds directly to the *vip*A promoter region in the T6SS gene cluster and positively regulates T6SS production and function. Moreover, we demonstrated that *fur* deletion causes significant impairments in interbacterial competition activity, adhesion, and virulence of *A. hydrophila in vitro* and *in vivo*. These findings suggest that Fur has a positive regulatory effect in T6SS function and increases the interbacterial competitive activity and pathogenicity of *A. hydrophila*.

## 2. Materials and methods

### 2.1. Plasmids, bacterial strains, cell line, and experimental fish

*Aeromonas hydrophila* strain GD18 was isolated from the diseased grass carp (*Ctenopharyngodon idella*) in 2017, which led to MAS in Guangdong Province of China (Li et al., 2021). All the bacterial strains, plasmids, and primers used in this study are listed in Tables 1, 2 (Kennedy et al., 1999; Kang et al., 2002; Liu et al., 2016). *Aeromonas hydrophila* strains GD18 and *E. coli* were grown in Luria Broth (LB) (pH 7.4) at 28°C and 37°C, respectively. When necessary, the antibiotic ampicillin (Amp; 100 μg/mL), chloramphenicol (Cm; 30 μg/mL), and kanamycin (Kan; 50 μg/mL) were supplemented in the medium. The Deferoxamine (200 μmol/mL) was added to the LB medium to prepare iron-depleted media.

**Table 1:** Strains and plasmids used in this study.

Strains and plasmids	Description	Source
Strains		
*Aeromonas hydrophila*		
GD18	Wild type	Lab collection
Δ*fur*	*fur* deletion mutant	This work
Δ*hcp*1/2	*hcp*1 and *hcp*2 double-deletion mutant	13
CΔ*fur*	Δ*fur* complemented with *fur* gene located in the plasmid of pMMB207	This work
*Escherichia coli*		
χ7213	*thr*-1 *leu*B6 *fhu*A21 *lac*Y1 *gln*V44 *rec*A1 Δ*asd*A4 Δ(*zhf*-2::Tn10) *thi*-1	23
Plasmids		
pRE112	Suicide vector, *sac*B, mob^−^(RP4)R6K ori, Cm^r^	24
pRE112-*fur*	pRE112 derivative, designed for knockout of *fur*, Cm^r^	This work
pMMB-207	Low-copy-number vector, Cm^r^	25
pMMB-*fur*	Plasmid pMMB207 carrying the complete ORF of *fur*, Cm^r^	This work
pMMB-*gfp*	Green fluorescent protein (GFP) expression, Cm^r^	This work

**Table 2:** Primers used in this study.

Primers	Sequence (5′-3′)	Target gene
p1	CGGGGTACCCCAACAAGCAGTTCAAGCGACTG	Upstream fragment of *fur*
p2	TGCGAATAAATAGAAATGTGATTGGCAGAACC	
p3	CGCGGGTTCTGCCAATCACATTTCTATTTAT	Downstream fragment of *fur*
p4	CGGGGTACCTCGAACTCGGGAAAGTGCTTGTCTC	
BamHI-gfp-F	CGGATCCATGAGTAAAGGAGAAGAACTTTTCACTGGAG	*gfp*
SacI-gfp-R	CGAGCTCTTATTTGTATAGTTCATCCATGCCATGTGTAATCC	
EcoRI-fur-F	CCCGAATTCATGGCAGACAACAACCAAGCGT	*fur*
XhoI-fur-R	CCCCTCGAGTCAATCGTCGTGCTTGCAGTCGC	
q16s-F	TTTAACCTTGCGGCCGTACT	16S
q16s-R	CCTGGACAAAGACTGACGCT	
qhcp-F	ACAAAGCCGTGCCGCTGATGT	*hcp*
qhcp-R	TGCGGCATCTGGCAGTCGAT	
qvgrG-F	TCGTGCGTATCCAGCATACC	*vgr*G
qvgrG-R	CAAGTGGTGCTGGCTTTCAC	
qvash-F	TGCTGCTCGGCATGCTGGAT	*vas*H
qvash-R	AAAACCGGCGTGCTCGATGC	
qtle1-F	AAGGCTGGGGCAAAGGCGTT	*tle*1
qtle1-R	TGCAGCACCACATCGACGCA	
qvipA-F	ATGATGGTGGTCGGCAACAT	*vip*A
qvipA-R	TCGTCCTGGCTGTTTTCCTC	

Healthy grass carp weighing 200 ± 20 g were bought from Xiantao Fish Hatchery, Hubei province, China. Wild-type AB strain zebrafish were obtained from the Institute of Hydrobiology of Chinese Academy of Sciences (Wuhan, China). Before intraperitoneal injection, the fish were acclimatized in the laboratory for 2 weeks. The animal experiment protocol was approved by the Laboratory Animal Monitoring Committee of Huazhong Agricultural University. All efforts were made to minimize suffering.

### 2.2. DNA and promoter sequence analyses

BLASTN and BLASTX (BLAST, basic local alignment search tool) were used to examine DNA sequences. Promoter identification was carried using the prokaryotic promoter prediction program tools, available at.^1^ The Fur binding box was searched using the T6SS genes’ 500-bp upstream sequence (from position −500 bp to 0 bp relative to the corresponding gene translation start site).

### 2.3. Construction and characterization of Δ*fur* mutants

Mutants were constructed using a previously described unmarked deletion method (Zhou et al., 2020). The primers were constructed using the gene sequence of *fur* in *A. hydrophila* GD18. Primer pairs p1/p2 and p3/p4 were used to amplify upstream and downstream flanking fur fragments, respectively. The primer pair p1/p4 was used in the fusion PCR. The PCR products amplified by the primer pair p1/p4 were introduced into the suicide plasmid pRE112, obtaining pRE-*fur*. Single-crossover mutants screened on Luria Agar (LA) media containing chloramphenicol. Allelic exchange was accomplished by culturing the single-crossover mutants on LA plates containing 7% sucrose. PCR using primers p1/p4 and direct DNA sequencing verified the Δ*fur*. The *fur* fragment was amplified from the wild type (WT) strain for gene complementation. The PCR product was cloned into pMMB207, resulting in pMMB-CΔ*fur*. The pMMB-CΔ*fur* was transconjugated into Δ*fur* strain to obtain the complemented strain CΔ*fur*. *hcp*1 and *hcp*2 double-deletion mutant Δ*hcp*1/2 as a T6SS- control strain was generated by our group (Li et al., 2021).

To detect the *A. hydrophila* strains growth kinetics, overnight cultures were diluted 1:500 and place in LB medium at 28°C with 200 rpm shaking. Hourly samples were taken, and optical densities were evaluated at 600 nm (OD_600_).

### 2.4. Lactate dehydrogenase cytotoxicity and adhesion assays

The LDH Cytotoxicity Assay Kit (Promega, USA) was used to quantify LDH release. *Ctenopharyngodon idella* kidney (CIK) cell line monolayers were infected with *A. hydrophila* at a multiplicity of infection (MOI) of 5 for 2 h, and supernatants were collected to evaluate LDH release. The cytotoxicity % was estimated using the manufacturer’s recommendations. LDH release from uninfected cells into the culture supernatant was represented by OD_490_ spontaneous, while LDH release acquired by lysis of uninfected cells was represented by OD_490_ maximal release.

The adhesion experiments were carried out in triplicate as described (Dou et al., 2018). The monolayers of CIK were grown for 24 h in a 12-well cell culture plate, and then infected with *A. hydrophila* at a MOI of 5. Non-adherent bacteria were eliminated by washing three times with PBS after two hours of incubation at 28°C and 5% CO_2_, and adhering bacteria were enumerated by plating on LA medium. Un-washed wells bacteria were enumerated as the total CFU after incubation. The adhesion rate was calculated by dividing the adhesion bacteria’s colony forming units (CFU) by the total CFU after incubation.

### 2.5. Bacterial infection

To assess the pathogenicity of the *A. hydrophila* strains, the median lethal dosage (LD_50_) was carried out in zebrafish as previously described (Dong et al., 2021). Briefly, each *A. hydrophila* strain was cultured overnight with LB medium, then overnight cultures were seeded into new medium at a rate of 1:100. After 5 h culture, the mid-logarithmic bacterial culture was washed and resuspended in sterile PBS and serially diluted tenfold from 10^2^ to 10^7^ CFU/ml. Six groups of 10 zebrafish per group were injected intraperitoneally (*i.p.*) with 10 μl of the bacterial solution in PBS for each *A. hydrophila* strain. Furthermore, as a negative control group, 10 zebrafish were injected just with PBS. The fish were monitored daily for 14 d. On 14 d after infection, surviving fish were euthanized. LD_50_ values were calculated according to *Karber’s* methods.

Bacterial loads in the blood, spleen, kidney, and liver were assessed for grass carp as described previously (Hu et al., 2020), with some modifications. At 24 h post-infection, grass carp *i.p.* infected with 2.73 × 10^3^ CFU/fish by *A. hydrophila* were euthanized and dissected. The target organs were collected, weighed, and homogenized with PBS to obtain supernatant. Using a 10-fold dilution method, blood and supernatant were plated on LB plates for number of bacterial.

### 2.6. Bacterial competition assays

Bacterial competition experiments were conducted between *A. hydrophila* and *E. coli* in accordance with previously described methods with minor modifications (MacIntyre et al., 2010). *Aeromonas hydrophila* GD18 and its derived strains were ampicillin resistant, and kanamycin resistance was conferred in *E. coli* DH5α after it was transformed with pET-28a (Kan^r^). The bacterial strains were cultured to OD_600_ of 0.5, and then the fresh attacker (*A. hydrophila*) and prey (*E. coli* DH5α) were combined at a 1:5 ratio in LB plates with nitrocellulose membranes for 3 h at 28°C. To select for prey bacteria, bacteria were harvested, diluted, and placed onto LB plates containing kanamycin. The prey strain’s survival rate was then determined. The experiment was carried out in triplicate.

### 2.7. Electrophoretic mobility shift assay and promoter activity analysis

To construct pET-23b-*fur*, the open reading frame of *fur* of *A. hydrophila* GD18 was amplified by PCR with the primer pair *fur* F1/R1, and the PCR products were ligated into pET-23b (see Table 2). The recombinant plasmid was transformed into *E. coli* BL21 (DE3). Protein expression in *E. coli* BL21(DE3) cells was induced with 0.5 mM isopropyl-β-D-thiogalactoside (IPTG) at 37°C for 6 h. Fur protein was affinity-purified using Ni-NTA resin column (BBI, China) as described previously (Cui et al., 2019). The purified protein was measured with Pierce BCA protein assay reagent (Thermo Fisher Scientific).

EMSA was performed using the EMSA kit (Thermo Fisher Scientific, USA) in accordance with the manufacturer’s instructions. Purified Fur protein (5.0 μg) was incubated in a 20 μL reaction with the biotin-labeled and purified oligonucleotide. The DNA/protein complexes were placed onto a 2% agarose gel, electrophoresed, and transported to a nylon membrane. A chemiluminescent substrate was used to detect the biotin-labeled DNA. Only DNA probes and only Fur protein were set as control.

We amplified the *vip*A gene promoter (from position bp −500 to +100 bp relative to the transcriptional start site) and cloned into the pMMB-207 vector harboring a GFP reporter to generate the transcriptional GFP fusion plasmid. The recombinant plasmid was transformed into the WT and Δ*fur* strains, respectively. Strains transformed with recombinant plasmids were harvested at the OD_600_ of about 1.0. The GFP activity was measured using a SpectraMax i3X (Tecan, Austria). The GFP activity was calculated as light units/OD_470_.

### 2.8. Real-time quantitative PCR

T6SS genes expression levels were examined using RT-qPCR as described in previous research (Wang et al., 2019). *A. hydrophila* were cultured for 12 h in LB medium or LB medium with Deferoxamine and the bacteria were harvested for RNA-extraction. TRizol reagent (Invitrogen, USA) was used to extract total RNA in accordance with the manufacturer’s instructions. Reverse transcription was conducted using the random primers within M-MLV reverse system (Promega, USA). A RT-qPCR reaction was performed with Fast SYBR Green PCR Master Mix (Bio-Rad, USA). The CFX96 Real-Time System (Bio-Rad, USA) was used to perform all reactions. Table 2 shows all the primers that were utilized. The 2^-ΔΔCT^ method was used to calculate gene expression. 16sRNA was employed as a reference gene. Relative expression level was shown as fold change relative to the level of the wild-type strain GD18 grown in LB medium. All of the qPCR experiments were conducted for at least three times.

### 2.9. Hcp protein secretion assay

Bacteria were cultured in LB medium or LB medium with Deferoxamine. After 12 h, a 0.22-μm filter (Millipore, USA) was used to collect and filter supernatants of LB bacterial culture. The bacteria were rinsed with PBS buffer and centrifuged at 10000 g for 10 min. Anti-Hcp and anti-GADPH (Abclonal, China), were used as primary antibodies. An HRP-goat anti-mouse or HRP-goat anti-rabbit IgG polyclonal antibody (Abclonal, China) was used as the secondary antibody. Blot images were captured in an Image Quant LAS 4000 (GE Healthcare, USA).

### 2.10. Statistical analysis

All statistical analyses were conducted using GraphPad Prism v 8.0 (GraphPad Software Inc., USA). Data are presented as mean ± standard deviation. A two tailed unpaired t test was used to compare independent group, and a *value of p* of <0.05 was considered statistically significant (*p < 0.05, **p < 0.01, ***p < 0.005, ****p < 0.0001).

## 3. Results

### 3.1. Fur positively regulates the expression of T6SS in *Aeromonas hydrophila*

**Figure 1:**
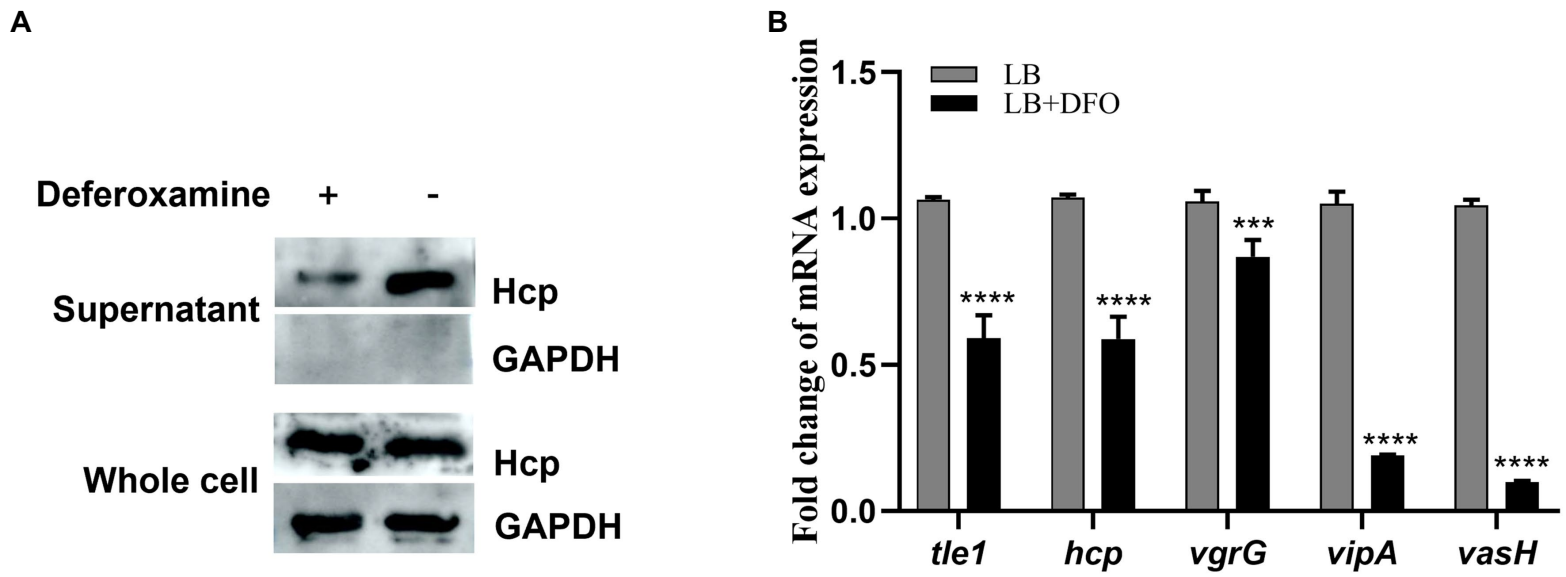
T6SS is repressed under iron-depleted conditions in *Aeromonas hydrophila*. **(A)** The expression and secretion of Hcp were analyzed by western blot. An anti-GAPDH antibody was used as control. **(B)** RT-qPCR analysis of the transcripts of the T6SS core genes in iron-depleted medium, presented relative to the expression in LB medium. The error bars represent mean ± standard deviations from three independent experiments (***p < 0.001; ****p < 0.0001).

Iron is one of the essential nutrients consumed by vertebrates as a defense mechanism against bacterial infection. This shortage is an environmental signal sensed by some pathogenic bacteria to modulate the expression of virulence genes. To investigate the response of theT6SS under iron-limiting conditions in *A. hydrophila*, Hcp expressed in the supernatant, as the indicator of functional T6SS was examined by western blot. The results showed that the secretion of Hcp decreased under iron-depleted conditions (Figure 1A). Consistent with this observation, RT-qPCR results showed varying degrees of down-regulation of T6SS core genes *hcp, vgr*G, *vip*A, and *vas*H (Figure 1B), indicating the depression of T6SS genes of *A. hydrophila* GD18 under iron-limiting conditions.

**Figure 2:**
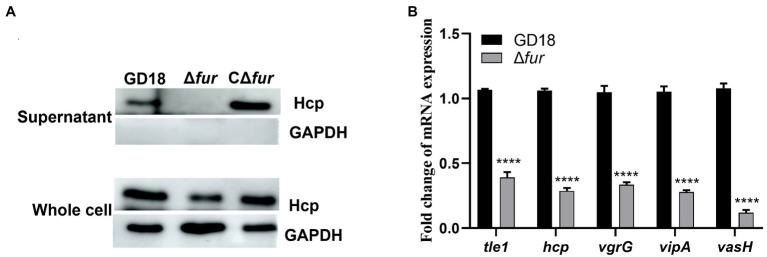
Fur positively regulates the expression of T6SS genes in *A. hydrophila*. **(A)** The expression and secretion of Hcp in different *A. hydrophila* strains grown in iron-replete medium were determined by western blot. An anti-GAPDH antibody was used as control. **(B)** RT-qPCR analysis of the transcripts of the T6SS core genes *tle*1, *hcp*, *vgr*G, *vip*A, and *vas*H in Δ*fur*, presented relative to the expression in wild-type strain. *A. hydrophila* strains grown in iron-replete medium. The error bars represent mean ± standard deviations from three independent experiments (****p < 0.0001).

Given that Fur has been implicated in the modulation of bacterial genes associated with environmental tolerance and virulence by binding to the Fur box (Porcheron and Dozois, 2015), we constructed the *fur* mutant and complemented strains to determine whether T6SS genes are potentially regulated by Fur in *A. hydrophila* GD18. Then, western blot was performed to further detect the production levels of Hcp in different strains. The secretion of Hcp protein in Δ*fur* was totally abolished, while complementation of the *fur* gene restored Hcp secretion (Figure 2A). The transcripts of the T6SS core genes were also measured by RT-qPCR. Consistent with the western blot results, inactivation of *fur* led to different degrees of down-regulation of T6SS core genes, including *tle*1 (2.6-fold), *hcp* (3.5-fold), *vgr*G (3-fold), *vip*A (3.6-fold), and *vas*H (8.4-fold; Figure 2B). Based on these findings, Fur appears to be a positive regulator of T6SS expression in *A. hydrophila*.

### 3.2. Fur protein binds directly to the promoter region of *vip*A in the T6SS gene cluster

**Figure 3:**
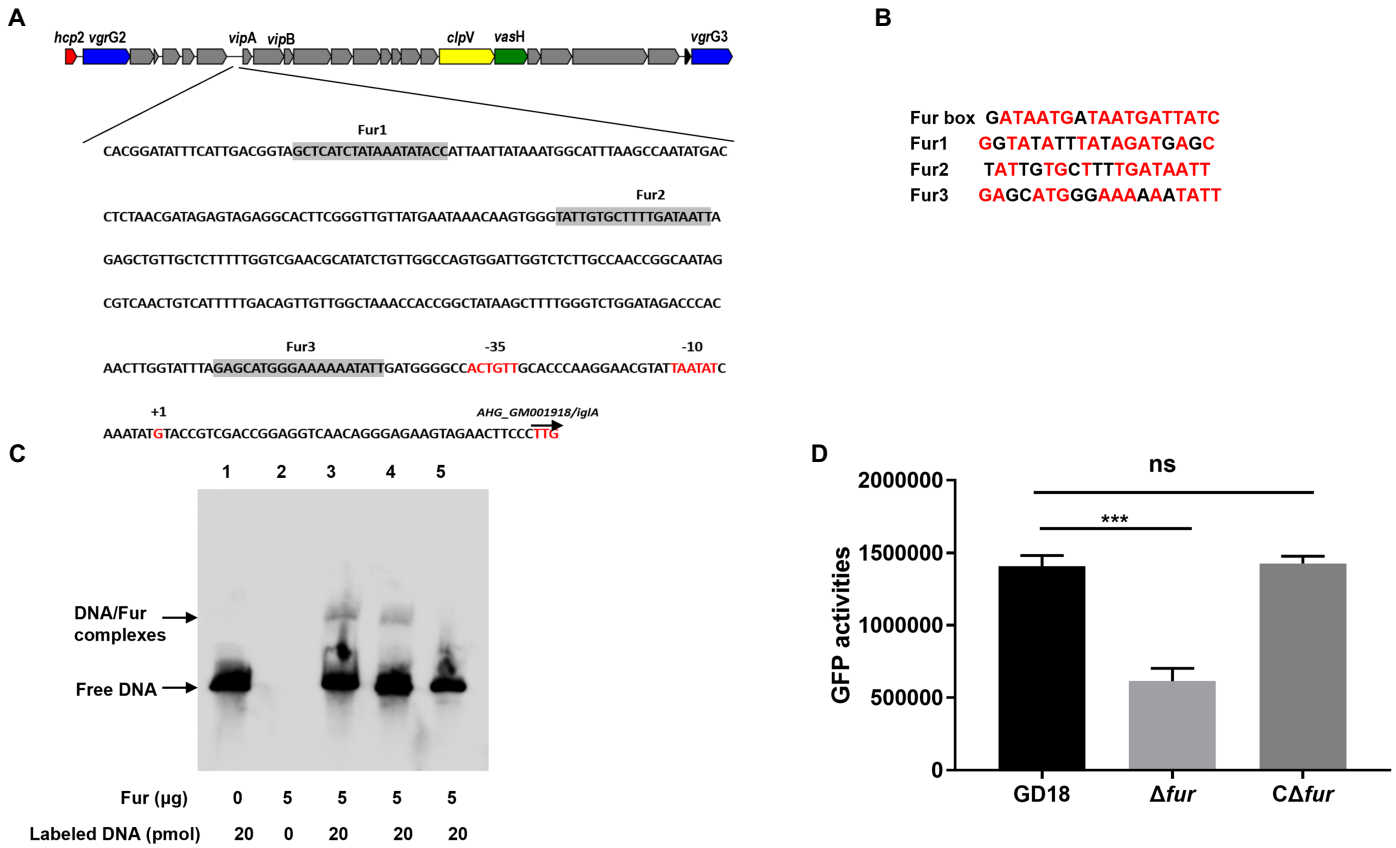
Fur protein binds directly to the promoter region of *vip*A in the T6SS gene cluster. **(A)** *In silico* analysis for Fur binding box prediction in the T6SS gene cluster. The putative Fur binding boxes are indicated as gray boxes. The transcriptional initiation site, the corresponding 10 and 35 boxes, and the translational start site are indicated in red letters. **(B)** Sequence alignment of these putative Fur binding boxes with the Fur box consensus sequence. Bases identical to the consensus are shown in red. **(C)** The different DNA fragments of *vip*A promoter region were amplified, biotin labeled, and used as probes. EMSA of Fur protein (5 μg) and biotin-labeled probes was performed. Line 1: DNA probes control; line 2: Negative control; line 3: Fur1; line 4: Fur2; line 5: Fur 3. **(D)** The GFP activities of the P*_vipA_*-*gfp* reporter fusions were measured in the WT and Δ*fur* strains. The error bars represent mean ± standard deviations from three independent experiments (***p < 0.001; ns, no significance).

To investigate the mechanism of Fur regulation of T6SS expression, we then examined the potential Fur binding boxes in the promoter regions of the whole T6SS gene cluster in *A. hydrophila* strain GD18 (Figure 3A). Three putative Fur-binding sites were predicted to locate upstream of *vip*A open reading frame. The three putative Fur binding boxes, named Fur1, Fur2, and Fur3, showed 63 to 84% homology to the consensus Fur binding site (Figure 3B). Fur1, Fur2, and Fur3 were present at positions −341 to −322 bp, −239 to −221 bp, and − 64 to −45 bp upstream of the *vip*A transcriptional start site, respectively (Figure 3A). No Fur binding box was found in other promoters of the T6SS gene cluster.

To validate whether Fur binds these Fur-binding sites upstream of *vip*A, EMSA was conducted using recombinant protein and the biotin-labeled PCR fragments encompassing the potential Fur boxes in the *vip*A promoter. Fur protein interacted with the Fur1and Fur2 probe but not with the *Fur3* probe (Figure 3C), suggesting that Fur binds straight to the promoter region of *vip*A in the T6SS gene cluster to activate the transcription of the genes in *A. hydrophila*.

To further verify this assumption, we constructed a GFP transcriptional reporter fusion plasmid in which GFP is governed by the *vip*A promoter, and then transferred it into the *A. hydrophila* strains to examine the regulatory effect of Fur on the promoter activities. The results indicated that the expression of GFP under the control of *vip*A promoter in Δ*fur* was reduced (56%) and complementation of fur restored expression of GFP to the WT strain level (Figure 3D). Overall, these results indicate that Fur positively regulates the expression of T6SS by binding straight to the *vip*A promoter in *A. hydrophila*.

### 3.3. Fur facilitates the interbacterial competition of *Aeromonas hydrophila in vitro*

**Figure 4:**
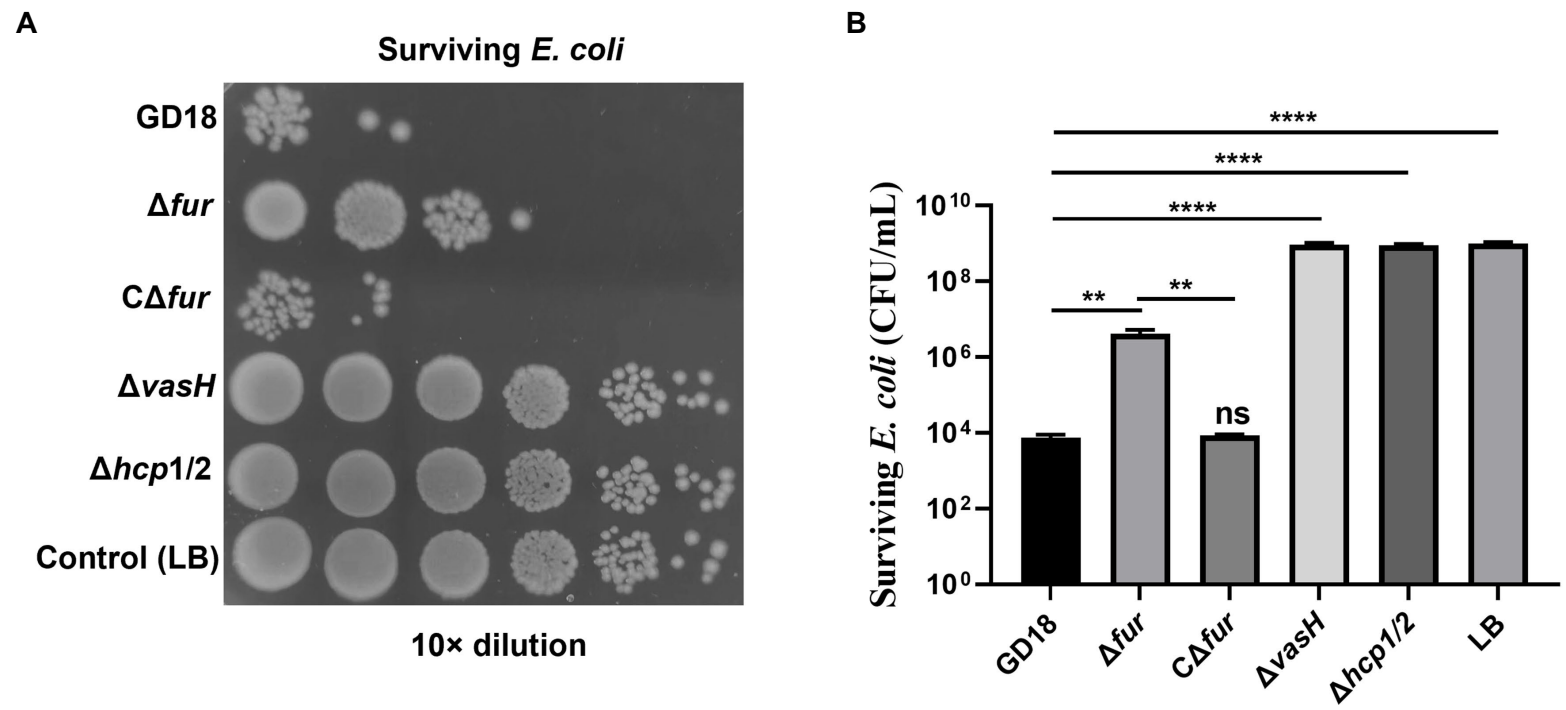
Fur facilitates the interbacterial competition of *A. hydrophila in vitro*. The survival of *Escherichia coli* was determined by detecting CFUs post incubation with different *A. hydrophila* strains as indicated. The fresh attacker (*A. hydrophila*) and prey (*E. coli* DH5α) were combined at a 1:5 ratio in LB plates with nitrocellulose membranes for 3 h at 28°C. **(A)** The surviving *E. coli* cells were serially diluted and determined on the LB plate containing kanamycin. Control (LB) indicates incubation of *E. coli* with sterile LB medium alone. Δ*hcp*1/2 mutant group was set as the T6SS defective control group. **(B)** The prey strain’s survival rate was then determined. The error bars represent mean ± standard deviations from three independent experiments (**p < 0.01; ****p < 0.0001).

Previous studies by our group and others supported *A. hydrophila* T6SS contributes to the interbacterial competition (Cui et al., 2019; Li et al., 2021). Thus, we further analyzed whether the antibacterial activity was influenced in the Δ*fur* strain. *E. coli* DH5α strain was taken as prey and *A. hydrophila* strains were taken as predators (Figure 4). As expected, after 3 h co-incubation with *A. hydrophila* WT strain, the surviving *E. coli* exhibited 7log_10_ reduction in comparison to *E. coli* cultured alone. The bactericidal capacity of Δ*fur* strain was markedly reduced (*p* < 0.01) and complementation of *fur* restored bactericidal activity to the WT strain level. Δ*hcp*1/2 was set as a T6SS-negative control, suggesting that the deletion of T6SS resulted in abolished bactericidal effect. In conclusion, the activation of Fur promotes the interbacterial competition of *A. hydrophila.*

### 3.4. Fur contributes to bacterial adherence and cytotoxicity *in vitro*

**Figure 5:**
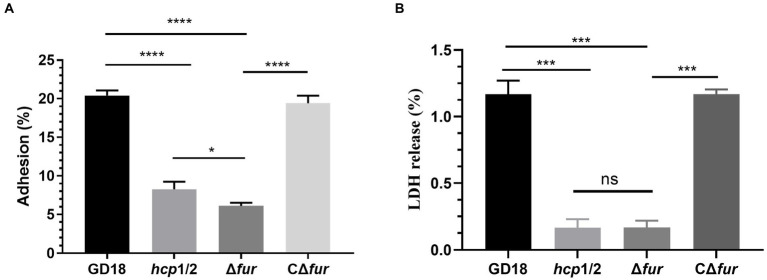
Fur contributes to cell adherence and cytotoxicity *in vitro*. **(A)** Adhesion rate of the WT and mutant strains. **(B)** Cytotoxicity results after 2 h of incubation. Δ*hcp*1/2 mutant group was set as the T6SS defective control group. The error bars represent mean ± standard deviations from three independent experiments (*p < 0.05; ***p < 0.001; ****p < 0.0001; ns, no significance).

The effect of Fur on bacterial adhesion and cytotoxicity to CIK cells was investigated. CIK cells were infected with the WT, Δ*hcp*1/2, Δ*fur*, and the complementation strain CΔ*fur* at 5 MOI. Firstly, the numbers of bacteria adhering to CIK cells were determined. The mutant strain Δ*fur* presented a 3.4-fold decreased adherence ability in comparison with that of the WT strain (*p* < 0.001; Figure 5A). Adhesion capacity was restored in the complementation strain CΔ*fur*. Interestingly, Δ*hcp*1/2, the T6SS-negative mutant strain exhibited stronger (1.4-fold) adherence capacity compared with Δ*fur* (*p* < 0.001).

The release of LDH in CIK cells was measured to analyze the cytotoxicity of *A. hydrophila* strains. Compared with the WT strain, Δ*fur* infection led to a significant decreased (90%) of LDH release after 2 h of infection (Figure 5B) and complementation of CΔ*fur* restored cytotoxicity to the WT level. Similarly, Δ*hcp*1/2 was also strongly outcompeted by the WT strain. The analysis revealed that bacterial adhesion and cytotoxicity of the mutant strain Δ*fur* were significantly decreased in comparison with those of the WT strain GD18.

**Figure 6:**
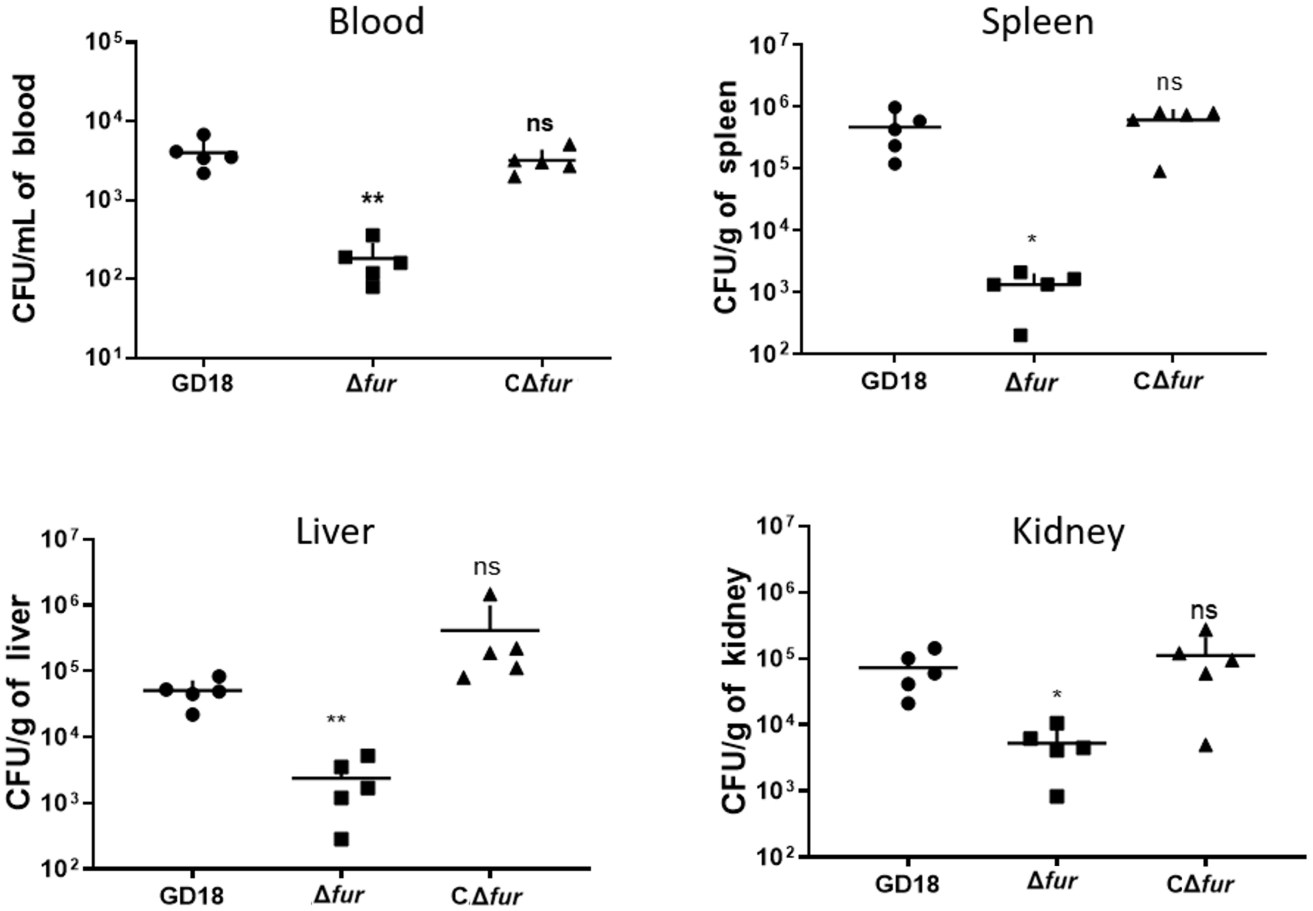
Colonization of grass carp tissues by WT strains and Δ*fur*. Grass carp *i.p.* infected with 2.73 × 10^3^  CFU/fish by *A. hydrophila* were euthanized and dissected 24 h post-infection. The error bars represent mean ± standard deviations from three independent experiments (*p < 0.05; **p < 0.01; ns, no significance).

### 3.5. Fur is essential for both the systematic infection and virulence of *Aeromonas hydrophila in vivo*

Fur family members are significant virulence determinants of many bacterial pathogens (Troxell and Hassan, 2013). Therefore, we determined the roles of Fur during systemic infection of *A. hydrophila*. The LD_50_ values of the WT strain GD18, Δ*fur*, and Δ*hcp*1/2 strains were 2.73 × 10^2^ CFU/fish, 1.00 × 10^4^ CFU/fish, and 5.62 × 10^3^ CFU/fish, respectively (Table 3). The Δ*fur* was attenuated in virulence with a 37-fold increase of LD_50_, while Δ*hcp*1/2 with a 21-fold increase.

**Table 3:** Calculations of LD_50_s of the *A. hydrophila* GD18 and mutant strains in zebrafish.

Dose of challenge CFU	Number of death/Total			Survival rate (%)		
	GD18	Δ*fur*	Δ*hcp*1/2	GD18	Δ*fur*	Δ*hcp*1/2
10^5^	10/10	10/10	10/10	0	0	0
10^4^	10/10	5/10	5/10	0	50	50
10^3^	9/10	0/10	2/10	10	100	80
10^2^	0/10	0/10	0/10	100	100	100
LD_50_^a^	2.73 × 10^2^	1.00 × 10^4^	5.62 × 10^3^			

a The LD50 was calculated according to Karber’s method.

To determine whether the reduced virulence was linked with failure in systemic spread, we assessed the bacterial loads of *A. hydrophila* strains in the blood, spleen, liver, and kidney of grass carp at 24 h post-infection. The results showed bacterial loads in the above organs of the Δ*fur* infected group were decreased by 95, 99, 94, and 93% respectively, compared to those of WT strain infected group (*p* < 0.05 or *p* < 0.01; Figure 6). The complementation strain CΔ*fur* displayed a similar capacity of systemic infection with the WT strain. These results indicated that Fur potentiates the systemic infection and virulence of *A. hydrophila in vivo*.

## 4. Discussion

Virulence gene expression need to be precisely modulated in order for bacteria to adapt and survive in various surroundings or host cells. T6SSs are widespread among gram-negative pathogens, and they are critical virulence determinants that participated in virulence and interbacterial competition (Cianfanelli et al., 2016). In *A. hydrophila* strains, the integrity of T6SS varied geographically, but all strains possess a complete type VI secretion system or remnants of the T6SS (Tekedar et al., 2019). Mutation of T6SS core genes lead to attenuated virulence and bactericidal activity (Wang et al., 2018; Tekedar et al., 2019; Li et al., 2021). T6SSs were demonstrated to be tightly regulated and induced by external conditions in many bacteria. As there are two oxidation states for iron, redox catalysis of iron is important for a variety of cellular processes, including respiration and DNA replication (Cassat and Skaar, 2013). Given that practically all pathogens demand iron, free Fe^2+^ is limited inside the host due to its usual sequestration by a variety of cellular processes (Cassat and Skaar, 2013). In this study, the supplementation of Deferoxamine led to significantly down-regulated expression and secretion of T6SS core protein Hcp in *A. hydrophila.* Hcp secretion is regarded to be a reliable indicator of T6SS function (Wang et al., 2018). In addition, decreased transcripts of other T6SS core genes were also detected. However, according to previous studies, facing the iron limit in the host, pathogens usually co-regulated iron uptake and virulence systems including T6SS synergistically to improve iron availability. For instance, iron-limiting circumstances increase transcription of the H2-T6SS gene cluster in *P. aeruginosa* (Sana et al., 2012). In *S. enterica* Serovar Typhimurium, the T6SS core gene *clp*V was significantly up-regulated in response to the iron-depleted condition (Wang et al., 2019). Therefore, our finding that T6SS in *A. hydrophila* was more activated under the iron-replete condition was contrary to the previous understanding from other pathogens.

Fur is a general transcription factor in bacteria that regulates intracellular iron homeostasis. Fur complexes with iron binds to the Fur box, high content in A/T rich region, and it subsequently participates in the transcriptional regulation of various genes (Fontenot et al., 2020). However, there is limited study revealing the regulatory mechanism of Fur on T6SS, compared to other regulatory networks. Here, we discovered that Fur positively regulates the expression of T6SS genes in *A. hydrophila.* As the secretion of Hcp protein was totally abolished in *fur* mutant strain, while complementation of the *fur* gene restored Hcp secretion. Extensive evidence suggests that Fur functions as a transcriptional regulator by binding to homologous locations on the promoter region of the targeted gene (Escolar et al., 1999). Indeed, we predicted three putative Fur binding boxes in the whole T6SS gene cluster, which all locate upstream of *vip*A gene. Among these binding sites, only Fur1 and Fur2 directly bound to Fur protein while Fur3 displayed no affinity to the Fur protein. Fur3 neighbors nearest to *vip*A transcription start sites. Using a P*_vipA_*-*gfp* reporter fusion, we confirmed that Fur positively regulates the transcription of *vip*A promoter. The T6SS components VipA/VipB form tube-shaped structures. VipA/VipB tubule contraction can stimulate the injection of putative Hcp tubules into target cells with the VgrG tip on top (Wang et al., 2017). A previous study showed that Hcp secretion in *V. cholerae* O1 strain requires the presence of VipA (Bröms et al., 2013). Consequently, the affected transcripts of *vip*A in Δ*fur* make the failure of Hcp secretion reasonable. Although Fur’s role as a repressor is well-established, new research suggests that it can also operate as an activator *via* three distinct mechanisms: (1) indirect activation through small RNAs, (2) binding at *cis* regulatory regions that promote the recruitment of the RNA polymerase holoenzym, and (3) removing or blocking DNA binding of a repressor of transcription, as an antirepressor (Wang et al., 2018). Our finding indicated the regulatory mechanism of Fur on T6SS in *A. hydrophila* belongs to the second category. So far, Fur binding boxes have been characterized in the promoter regions of the T6SSs in *P. aeruginosa, S.* Typhimurium, enteroaggregative *E. coli* (EAEC), and *E. tarda*, and the Fur proteins all act as negative regulators (Brunet et al., 2011; Chakraborty et al., 2011; Sana et al., 2012; Ma et al., 2018; Wang et al., 2019; Brunet et al., 2020). Therefore, to our knowledge, this was the first time that Fur protein was proved to work as an activator on T6SS by direct binding to the Fur boxes.

For *V. cholerae*, *P. aeruginosa*, EAEC, and *Salmonella*, pathogens use T6SSs as antibacterial weapons to compete efficiently within specific niches. Therefore, we tried to determine whether Fur-mediated T6SS regulation modulates *A. hydrophila* competition toward other microorganisms. Our results demonstrated that the bactericidal ability of the mutant strain Δ*fur* was significantly weakened, but still stronger than the T6SS-abolished strain Δ*hcp*1/2. The T6SS delivers effectors into target cells by employing a contractile sheath to propel an inner tube into neighboring target cells, similar to an inverted bacteriophage tail (Dong et al., 2015). Thus, on one hand, the lack of Fur protein as an active regulator on T6SS in Δ*fur* certainly affected the secretion of related bactericidal effectors. On the other hand, we detected 2.6-fold down-regulation of *tle*1 transcripts in Δ*fur*. Tle1^AH^, identified as an effector protein of T6SS in *A. hydrophila* NJ-35, mediates interbacterial antagonism (Ma et al., 2020). As *tle*1 does not locate in the T6SS gene cluster in GD18 strain, the regulatory mechanism of Fur on its transcription needs further study.

The link between Fur and pathogenicity has been the subject of extensive research in recent years. In host–microbe interactions, Fur regulates the expression of virulence factors, as well as toxin production in free-living and environmental bacteria (Fillat, 2014). In *E. tarda*, Fur suppresses *eth*B, encoding the activation/secretion apparatus of the haemolysin system (Chakraborty et al., 2011). In *Staphylococcus aureus*, mutation of *fur* lead to increasing of cytotoxic potential to different human epithelial and promyelocytic cells (Torres et al., 2010). Fur has the ability to suppress toxin or haemolysin synthesis in some bacterial species while also being able to stimulate it in others (Porcheron and Dozois, 2015). In this study, Fur was found to contribute to cell adherence and cytotoxicity *in vitro* and is necessary for the systemic infection and virulence of *A. hydrophila in vivo*. The mutation of *fur* reduced the pathogenicity of *A. hydrophila* by 37-fold. This was consistent with the previous study that the *fur* knockout mutant shows significantly reduced pathogenicity compared to the wild-type parental strain in *A. salmonicida* (Ebanks et al., 2013). Moreover, infection studies of catfish (*Ictalurus punctatus*) and Japanese flounder (*Paralichthys olivaceus*) using *fur* deletion mutants of *E. ictaluri* and *P. fluorescens*, respectively, show that these genetically engineered pathogens could be useful tools in the development of immersion-oral vaccines for the fish industry (Wang et al., 2009; Santander et al., 2012). Compared to their drastic attenuation (1000-fold), Δ*fur* constructed in this study is still virulent, but has the potential to be genetically modified as a vaccine candidate.

**Figure 7:**
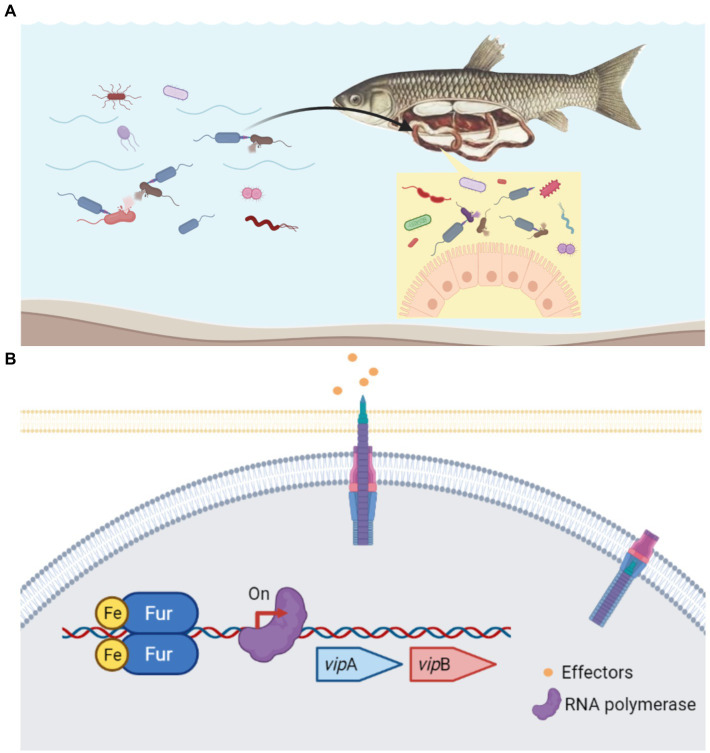
Schematic diagram of the Fur-mediated regulation of T6SS in *A. hydrophila*. **(A)** In the natural aquatic habitat or gastrointestinal tract of the host, *A.hydrophila* constantly has the problem of competing with other bacteria for niche under iron-replete conditions. **(B)** Under iron-replete conditions during infection, Fur binds in the promoter region of the T6SS *vip*A gene and promote the transcription. Fur protein in *A. hydrophila* could be a proper regulator to sense the iron concentration and work as the activator to regulate T6SS to compete with other microorganisms.

Aeromonads are not only prevalent environmental bacteria, but they can also colonize humans and animals quickly and cause opportunistic infections. *Aeromonas* spp. has a unique ability to colonize the guts of animals, including leeches, fish, cows, and humans, as well as a variety of other hosts (Lamy et al., 2021). Gastroenteritis is a typical symptom in MAS-affected fish. Free Fe^2+^ is normally limited inside the host since it is required in numerous biological processes as metalloprotein components and as cofactors or structural elements for enzymes (Porcheron et al., 2013). However, there is plentiful free Fe^2+^ in the lumen of the intestine, where the majority of dietary iron is absorbed (Conrad and Umbreit, 2002). Coincidentally, in the nutrient-rich environment of intensive fish farming, free iron is most likely available to *A. hydrophila* (Pajuelo et al., 2016). Along these lines, whether in the natural aquatic habitat or gastrointestinal tract of the host, *A. hydrophila* always faces the challenge of competing with other bacteria for niche under iron-replete conditions (Figure 7A). According to our study, Fur protein in *A. hydrophila* could be a proper regulator to sense the iron concentration and work as the activator to regulate T6SS to compete with other microorganisms (Figure 7B). In EAEC, *sci1* T6SS gene cluster is under the delicate regulation involving overlapping Fur and DNA methylation in different iron conditions (Brunet et al., 2011). As an opportunistic pathogen, *A. hydrophila* must have evolved successful survival mechanisms to adapt to changeable habitats. Thereby, more cross-regulatory mechanism of T6SS awaits to be resolved.

In summary, our findings identify a previously unrecognized role of Fur in promoting interbacterial competition and infection of *A. hydrophila* by binding and positively regulating the expression and function of T6SS. Iron is rich in the natural aquatic environment or the gastrointestinal tract of the host. Therefore, the positive regulation of the expression of T6SS by Fur represents an unusual strategy for *A. hydrophila* under iron-rich conditions to facilitate the T6SS-mediated bacterial dominance and virulence. Taken together, these results further elucidate the mechanisms by which multiple regulatory networks corporate, contributing to understanding gene expression for bacterial fitness and virulence.

## Data availability statement

The original contributions presented in the study are included in the article/supplementary material, further inquiries can be directed to the corresponding authors.

## Ethics statement

The animal study was reviewed and approved by Laboratory Animal Monitoring Committee of Huazhong Agricultural University.

## Author contributions

Y-AZ and YZ: conceived and designed the experiments, writing—review and editing. JL, ZW, and YH: performed the experiments. JL and ZW: data curation. JL and YZ: writing—original draft. All authors contributed to the article and approved the submitted version.

## Funding

The study was supported by the Laboratory of Lingnan Modern Agriculture Project (NT2021008), China Agriculture Research System of MOF and MARA (CARS-46), the National Natural Science Foundation of China (32073022), and HZAU-AGIS Cooperation Fund (SZYJY2021027).

## Conflict of interest

The authors declare that the research was conducted in the absence of any commercial or financial relationships that could be construed as a potential conflict of interest.

## Publisher’s note

All claims expressed in this article are solely those of the authors and do not necessarily represent those of their affiliated organizations, or those of the publisher, the editors and the reviewers. Any product that may be evaluated in this article, or claim that may be made by its manufacturer, is not guaranteed or endorsed by the publisher.

## Footnotes

^1^ http://www.fruitfly.org
